# The complete chloroplast genome of *Actinidia macrosperma*

**DOI:** 10.1080/23802359.2019.1692733

**Published:** 2019-11-21

**Authors:** Yi Chen, Yiqing Xu, Kai Zhang, Yanlin Song, Qiuyu He, Qiuyu Qian, Jian Xu

**Affiliations:** a Sun Nanjing Automatic Equipments Co. Ltd., Nanjing, China;; b College of Information Science and Technology, Nanjing Forestry University, Nanjing, China;; c Nanjing Visiable Information Technology Co. Ltd., Nanjing, China;; d Shandong Agriculture and Engineering University, Jinan, China;; e Nanjing XiaoZhuang University, Nanjing, China;; f Nanjing Muzhou Software Co. Ltd., Nanjing, China

**Keywords:** *Actinidia macrosperma*, chloroplast genome, assembly, phylogenetic analysis

## Abstract

*Actinidia macrosperma* (*A. macrosperma*) is a medicinal plant in China, which has been well known for its activities against leprosy and cancers. In this study, we assembled and characterized the complete chloroplast (cp) genome sequence of *A. macrosperma* in an effort to provide genomic resources for promoting its conservation. The cp genome is 156,231 bp in length, containing a pair of 23,720 bp inverted repeat (IR) regions, which is separated by a large single copy region (LSC) of 88,214 bp and a small single copy region (SSC) of 20,577 bp. A total of 132 genes were annotated in this cp genome, including 85 protein-coding genes, 39 tRNA genes, and 8 rRNA genes. Phylogenetic analysis highly supported that *A. macrosperma was* evolutionarily close to another *Actinidia* species *Actinidia deliciosa*.

As a traditional medicine, *Actinidia macrosperma* is commonly known as ‘ginseng of cat’ in China, because cats are sensitive to the volatile chemicals released from its aerial parts (Lu et al. 2007). It is a deciduous scandent shrub with orange fruits and white flowers. *Actinidia macrosperma* is endemic to eastern China, and mainly distributed in Zhejiang, Jiangsu, Jiangxi, and Hubei Provinces. In the folks of China, its root and stem have been used to treat leprosy, lung cancer, and esophageal cancer (Lu et al. 2007). Due to the enormous demand, the wild resource of this species has decreased sharply, and even become extinct. Thus, great attention should be paid to the reasonable development and effective protection of *A. macrosperma*. In this study, we assemble and characterize the complete cp genome of *A. macrosperma*, which will provide important genomic resources for developing optimum conservation and management strategies.

Total genomic DNA was extracted from fresh leaves of *A. macrosperma* sampled in April 2016 from an experimental orchard of Nanjing Forestry University (32°04′51″N, 118°48′46″E) using the DNeasy plant Mini Kit (Quiagen, Carlsbad, CA) (Tang et al. 2019). A voucher specimen was deposited in the Herbarium of Nanjing Forestry University (No. NF20160521). Purified DNA was then fragmented to construct an Illumina paired-end library, and then sequenced using the Illumina Hiseq 2000 platform. The raw sequencing data were filtered and trimmed by fastp program (Chen et al. 2018), and then fed into NOVOPlasty (Dierckxsens et al. 2017) for assembly using the *rbcL* gene and whole-genome sequence of *Actinidia deliciosa* (GenBank accession: NC_026691.1) as the seed sequence and reference genome, respectively. The assembled genome sequence was then annotated using Plastid Genome Annotator (PGA) (Qu et al. 2019) against the cp genome of *Actinidia tetramera*. The annotation result was inspected using Geneious v8.0.4 (Matthew et al. 2012) and adjusted manually as needed. The annotated cp genome was submitted to GenBank under accession number MN520000.

The circular cp genome of *A. macrosperma* was 156,231 bp (GC Content: 37.25%) in length, including a LSC region of 88,214 bp (GC Content: 35.53%), a SSC region of 20,577 bp (GC Content: 31.01%), and a pair of IR regions of 23,720 bp (GC Content: 43.17%). The cp genome of *A. macrosperma* encodes a total of 132 predicted functional genes, including 85 protein-coding genes, 39 tRNA genes, and 8 rRNA genes. Eleven of the protein-coding genes and six of the tRNA genes contain introns, 15 of which contain a single intron, whereas one protein-coding gene (*ycf3*) contains two introns.

The Maximum-Likelihood (ML) tree was constructed based on 76 conserved protein-coding genes of 19 other plant cp genomes using MEGA 6.0 with 1000 bootstrap replicates. We found that *A. macrosperma* was highly support to cluster with another *Actinidia* species with 100% bootstrap values, and the *Actinidia* genus was evolutionarily close to the Solanaceae plant (Figure 1). The complete cp genome sequence of *A. macrosperma* will provide a useful resource for its reasonable development and effective protection.

**Figure 1.**
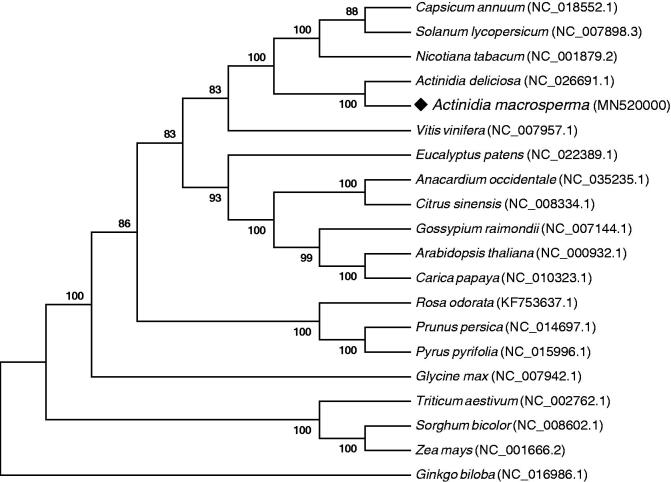
The Maximum-Likelihood phylogenetic tree of 20 plant chloroplast genomes based on 76 conserved protein-coding genes. Bootstrap values are listed for each node. GenBank accession numbers are listed right to the scientific names.
